# A case of Wilson’s disease combined with intracranial lipoma and dysplasia of the corpus callosum with review of the literature

**DOI:** 10.1186/s12883-024-03541-2

**Published:** 2024-01-25

**Authors:** Liangjie Zhang, Ling Zhu, Chunling Ci, Wenlong Ai, Yu Wang, Xun Wang

**Affiliations:** 1 Anhui University of Traditional Chinese Medicine, Hefei, 230012 China; 2 Department of Neurology, Affiliated Hospital of Neurology Research Institute, Anhui University of Traditional Chinese Medicine, Hefei, 230061 China; 3 Department of Neurology, Yueyang Hospital of Integrated Traditional Chinese and Western Medicine, Shanghai University of Traditional Chinese Medicine Shanghai, Shanghai, 201203 China

**Keywords:** Wilson’s disease, Corpus callosum dysplasia, Intracranial lipoma, Case report

## Abstract

**Background:**

Wilson’s disease (WD) is an inherited disorder of copper metabolism. Agenesis of the corpus callosum is the complete or partial absence of the major united fiber bundles connecting the cerebral hemispheres. Intracranial lipoma is an adipose tissue tumor resulting from an abnormal embryonic development of the central nervous system. The simultaneous occurrence of these three disorders is rare and has not been reported. This report focuses on the pathogenesis and association between the three disorders and highlights the importance of recognizing and effectively managing their coexistence.

**Case presentation:**

The purpose of this study was to present a patient with coexisting WD, intracranial lipoma, and corpus callosum dysplasia. We reviewed a female patient hospitalized in 2023 with clinical manifestations of elevated aminotransferases and decreased ceruloplasmin, as well as genetic testing for an initial diagnosis of Wilson’s disease. Subsequently, a cranial MRI showed corpus callosum dysplasia with short T1 signal changes in the cerebral falx, leading to a final diagnosis of Wilson’s disease combined with intracranial lipoma and corpus callosum dysplasia. The patient’s WD is currently stable after treatment with sodium dimercaptosulfonamide (DMPS) and penicillamine, and the patient’s abnormal copper metabolism may promote the growth of intracranial lipoma.

**Conclusion:**

The pathogenesis of WD combined with intracranial lipoma and corpus callosum dysplasia is complex and clinically rare. The growth of intracranial lipomas may be associated with abnormal copper metabolism in WD. Abnormal copper metabolism affects lipid metabolism and triggers inflammatory responses. Therefore, early diagnosis and treatment are beneficial for improvement. Each new case of this rare co-morbidity is important as it allows for a better assessment and understanding of these cases’ more characteristic clinical manifestations, which can help estimate the course of the disease and possible therapeutic options.

**Supplementary Information:**

The online version contains supplementary material available at 10.1186/s12883-024-03541-2.

## Introduction

Wilson’s disease (WD) is a rare autosomal recessive disorder with a global prevalence of approximately 1:30,000–1:50,000 [1]. It is caused by a mutation in the ATP7B gene in hepatocytes, leading to inactivation of the ATP7B transporter protein, which results in blocked copper excretion from the bile and a disturbance of the dynamic copper homeostasis [2]. Clinical damage to organs, including the liver, brain, and kidneys, was frequently observed, and some patients exhibited anomalous lipid metabolism [3]. Intracranial lipomas are extremely rare tumors, accounting for about 0.1 - 0.5% of all intracranial tumors[4], and were first described by Rokitansky in 1856 [5]. Intracranial lipomas may originate from abnormal differentiation of the primitive meninges around normally developing brain tissue, in which the meningeal tissue is gradually transformed into adipose tissue. However, the specific etiology of intracranial lipomas has not been clarified. Potential risk factors include genetics, chromosomal abnormalities, intrauterine infections, ischemia, and abnormal environmental exposures.

Furthermore, 30% - 50% of intracranial lipomas occur around the corpus callosum, while 55% - 75% occur in the midline region [4]. More than half of the intracranial lipomas are often associated with other lesions, including corpus callosum agenesis. Corpus callosum agenesis is a fetal developmental abnormality characterized by incomplete formation or partial absence of the corpus callosum and is one of the most common brain malformations. Although its incidence is relatively low, occurring in approximately 1:4,000 live births, it causes a wide range of clinical conditions [6]. Its independent existence was first reported in 1912 by Reil [7]. Although intracranial lipomas combined with corpus callosum dysplasia have been reported, no cases of all three disorders occurring together in patients with WD have been reported. Here, we report a case of a patient with WD who had both lipoma at the cerebral falx and corpus callosum dysplasia.

## Case presentation

**Fig. 1:**
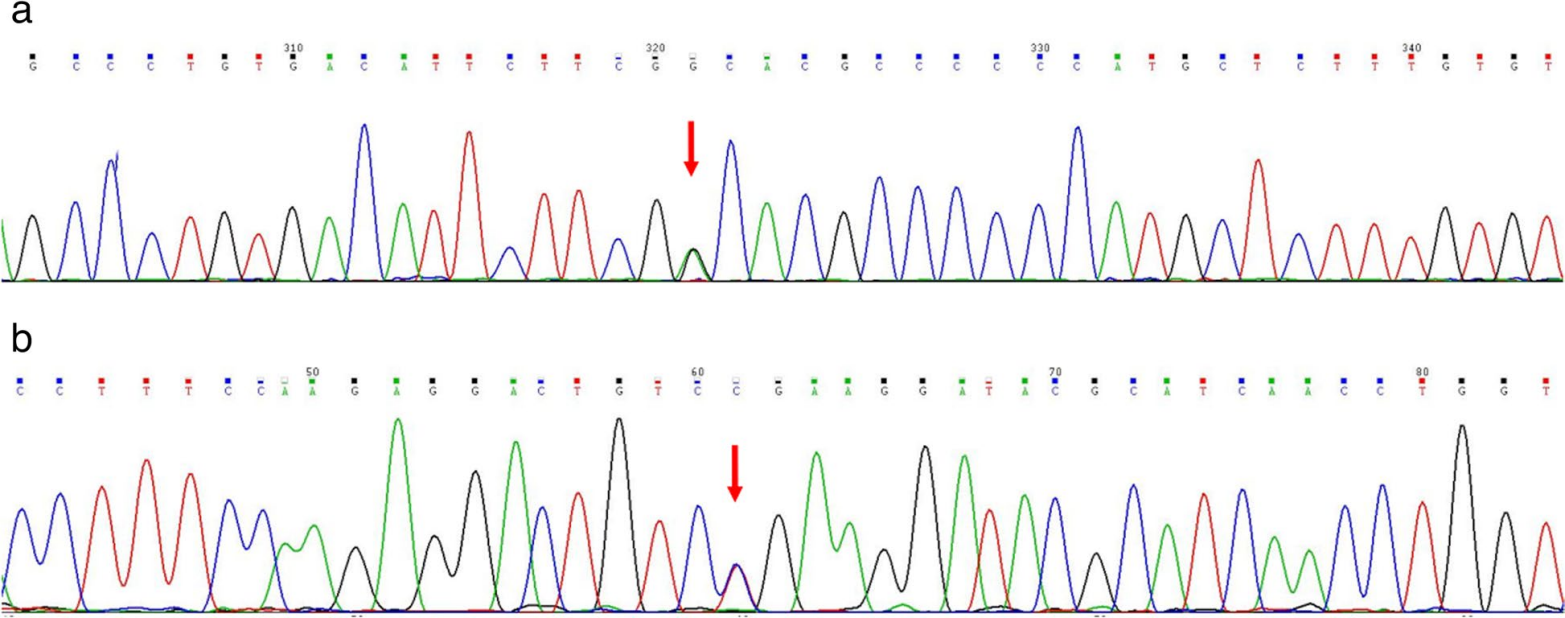
Mutation analysis of 21 exons and flanking sequences of ATP7B gene by applying targeted sequencing technology, verified using the sanger sequencing method. The results show that compound heterozygous missense variants c.2294A>G (p.D765G) (Figure1a) and c.3955C>T (p.R1319X) (Figure1b) exist in the ATP7B gene of the pre-witnesses, and all of them are known to be pathogenic mutations. Lineage validation revealed that the variants originated from the father (c.2294A>G) and mother (c.3955C>T), respectively

At the age of 9 years, the child’s physical examination showed that “liver function showed that alanine aminotransferase was 130 U/L and glutamic aminotransferase was 57 U/L”. Further examination showed that “ceruloplasmin was < 0.1 g/L (reference value 0.2-0.6 g/L)”, which is considered “Wilson’s disease”. At the age of 10 years, he was examined in our hospital for ceruloplasmin: 46.7mg/L (reference value 200.0-420.0mg/L); serum copper: 3.29umol/L (10.50-24.40umol/L); Copper oxidase: 0.038 OD (reference value > 0.200 OD), corneal K-F ring (+), and urinary copper 943.57ug 24 hours prior to medication. Family history is negative. ATP7B gene showed two compound heterozygous missense variants from parents (see Figure 1). The patient's head circumference and intercanthal distance were 48 cm and 4.5 cm, respectively. The physical examination showed no positive signs, and the Leipzig score was 10 [8]. The diagnosis was WD (preclinical).

**Fig. 2:**
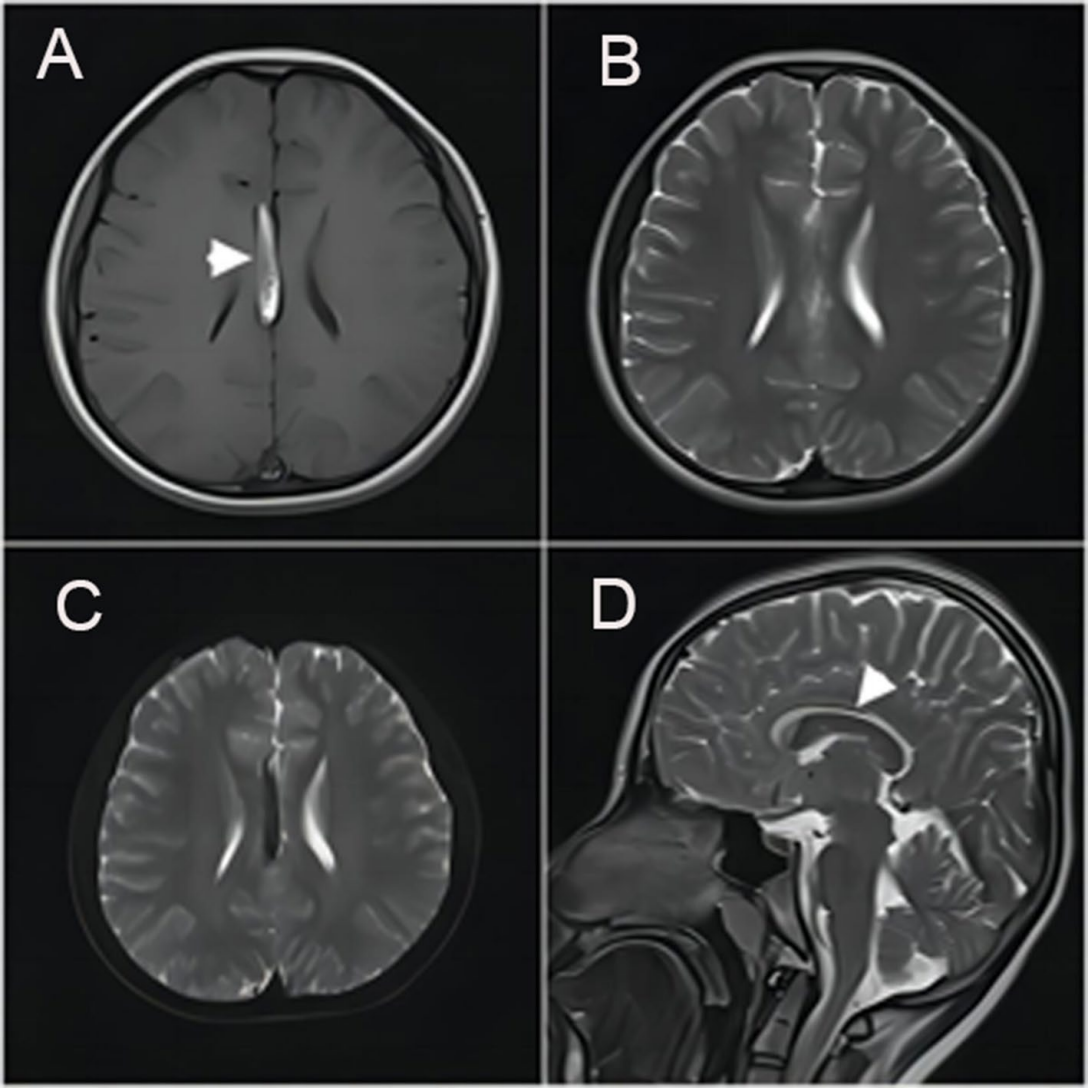
**A**, **B** are MRI scanning axial T1 and T2 images, respectively, which show parallel separation of bilateral lateral ventricles, widening of the spacing between the bodies of bilateral lateral ventricles, and a lipoma (white arrowhead) at the lower margin of the cerebral falx, with a size of about 40mm*9mm, with high signal in T1 and T2. **C** is an axial DWI image; the lesion is seen without diffusion restriction. **D** is a sagittal T2 image; the septum pellucidum is not closed, and the corpus callosum is thinned in the sagittal position (white arrow)

He was hospitalized and administered sodium dimercaptopropanesulfonate (250mg/d) for intravenous copper-repellent treatment, with a mean 24-hour urinary copper value of 780ug. He was discharged from the hospital and 125mg penicillamine(Three times a day) combined with 250mg DMPS (Twice a day) for oral alternating therapy, during which his condition was controlled steadily. At the age of 12 years, he was reexamined for ceruloplasmin: 31.3mg/L; serum copper: 3.39umol/L; and copper oxidase: 0.032 OD. The results of liver function indicators and other laboratory indicators are as follows (see Table 1): corneal K-F ring (+) and urinary copper 1531.59ug/24h. A cranial MRI (age: 12 years) showed a lipoma, thinning of the corpus callosum, and septum lucidum not closed (see Figure 2).

**Table 1:** The patient was admitted twice for examination and blood test results

Detection time	AST	ALT	Liver pSWE elastography measurements	TG	AFP
10 years old	74U/L	115U/L	1.68m/s moderate fibrosis	1.52mmol/L	14.9U/mL
12 years old	50U/L	100U/L	1.20 Mild fibrosis	1.69mmol/L	3.7U/mL

The normal range of indicators: AST(0-50 U/L); ALT (0-50/L); liver fibrosis staging and pSWE quantitative reference values: absent/mild fibrosis (0-1.34m/s); moderate fibrosis (1.34-2.2m/s); severe fibrosis (above 2.2m/s); TG (0.56-1,47mmol/L); AFP (< 10.0IU/mL)

## Discussion

Intracranial lipomas are congenital disorders resulting from abnormal differentiation of meningeal tissue during embryonic development. Most lipomas are small, usually pea-sized, and asymptomatic. They are often discovered incidentally on neuroimaging or only detected at autopsy, with incidence rates of 0.08% and 0.46%, respectively [9]. It has been shown that the protomembrane contains primitive perivascular reticular endothelium, cells specialized in storing fat. These abnormalities in the primitive cells of the meninges may ultimately allow them to differentiate directionally into adipose tissue and form lipomas. Thus, meningeal lipomas are not true tumors but an aberrant developmental process [9]. Intracranial lipomas are often accompanied by intracranial or extracranial malformations, with common comorbid malformations including hypoplasia of the corpus callosum, defect of septum pellucida, and craniosynostosis or agenesis. Interhemispheric lipomas interfere with the embryonic development of the brain, during which the corpus callosum is formed by the migration and attachment of nerve cells from midline structures, which may be the mechanism by which intracranial lipomas cause corpus callosum dysplasia. An intracranial lipoma may interfere with the normal cell migration and connections during these developmental processes, resulting in corpus callosum dysplasia. The patient in our case had a combination of corpus callosum dysplasia and an unclosed septum pellucidum [10], consistent with the above theory.

The patient, in this case, was diagnosed with WD and had a concurrent intracranial lipoma.WD is caused by defects in the ATP7B gene, resulting in abnormal copper metabolism, tissue copper accumulation, and copper-induced oxidative damage [11].Typical cranial MRI changes in WD usually present as symmetrical, high, or mixed signals on T2-weighted images, affecting mainly the putamen, pontine, midbrain, and thalamus [12, 13]. Although previous studies have less frequently addressed corpus callosum abnormalities, a study by Trocello et al. demonstrated that some patients with Wilson’s disease also have abnormalities of the corpus callosum [14]. The growth of intracranial lipomas may be associated with abnormal copper metabolism in WD. Abnormal copper metabolism affects lipid metabolism and triggers an inflammatory response. Abnormalities of lipid metabolism in a rat model of hepatic copper overload were demonstrated by Medici et al [15]. First, liver damage in patients with WD affects lipid metabolic pathways, such as the sphingolipid and glycerophospholipid metabolic pathways, leading to elevated serum concentrations of several lipid molecules which may promote the growth of intracranial lipomas [16, 17]. In addition, WD is accompanied by a certain degree of inflammatory response. Several studies have found that serum inflammatory factors such as interleukin-6(IL-6) and tumor necrosis factorα(TNF-α) are abnormally high in patients with WD. This phenomenon is attributed to the accumulation and abnormal metabolism of copper in the body, which leads to oxidative stress and cellular damage, triggering inflammatory responses and thus increasing the serum levels of various inflammatory factors [18]. These inflammatory factors may promote adipocyte proliferation, inhibit adipocyte apoptosis, and promote fat synthesis, leading to the growth of intracranial lipomas [19, 20]. Therefore, maintaining good liver function and keeping appropriate serum copper levels are essential to inhibit the growth of intracranial lipomas in patients [21]**.** In addition, Schaefer reported that the formation of subcutaneous lipomas in patients with WD may be associated with mutations in an adjacent region of the ATP7B gene [22]. Unfortunately, the patient in this case did not undergo relevant genetic testing.

There are extremely limited reports on the coexistence of WD, corpus callosum dysplasia, and intracranial lipoma in this case. The patient’s small head circumference and wide eye spacing may be related to corpus callosum dysplasia, which does not require special treatment. In addition, the patient’s intracranial lipoma was small and clinically insignificant, so no specific treatment was required, and only regular imaging monitoring was required. Only when the intracranial lipoma enlarges and is accompanied by symptoms such as convulsions, mental retardation, pituitary-hypothalamic endocrine disorders, and visual disturbances does targeted treatment need to be considered [23]. Currently, patients only need to be treated for WD to avoid copper’s high effects on the growth of intracranial lipomas [24]. In addition, patients need to follow a low-fat diet [25] because there is a correlation between significant lipoma growth and increased body fat content [26].

## Conclusions

Combining WD with intracranial lipoma and corpus callosum dysplasia is exceedingly rare. This case provides some evidence about the association between WD and both. However, further case studies and basic research are required to understand the mechanisms and relationships between them better.

### Supplementary Information

**Additional file 1.**

## Data Availability

No dataset was generated or analyzed during this study.

## Abbreviations

DMPS: Sodium Dimercaptosulphon
WD: Wilson’s disease
AST: Aspartate aminotransferase
ALT: Alanine aminotransfease
TG: Triglyceride
AFP: alpha-fetoprotein
