# Supplementary material for: A case of Wilson’s disease combined with intracranial lipoma and dysplasia of the corpus callosum with review of the literature
Source: BMC Neurol. 2024 Jan 25;24:44. doi: 10.1186/s12883-024-03541-2 (PMC10809563; doi:10.1186/s12883-024-03541-2)
Supplement: Supplementary file 1 — Additional file 1. [file 12883_2024_3541_MOESM1_ESM.docx]

| Screening for exonic mutations in the ATP7B gene |
| --- |
| Sequencing of exon 8 (EXON8) |
| 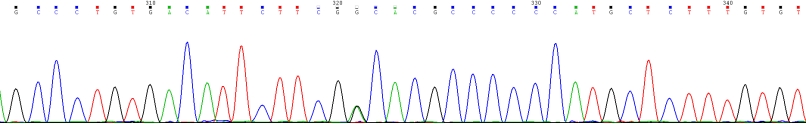  Sequencing of exon 19 (EXON19) |
| 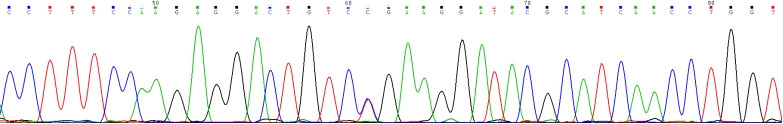  Subjects were detected with p.D765G (c.2294A>G) heterozygous mutation in exon 8 (EXON8) and p.R1319X (c.3955C>T) heterozygous mutation in exon 19 (EXON19). |
| Sequencing of exon 8 (EXON8) |
| 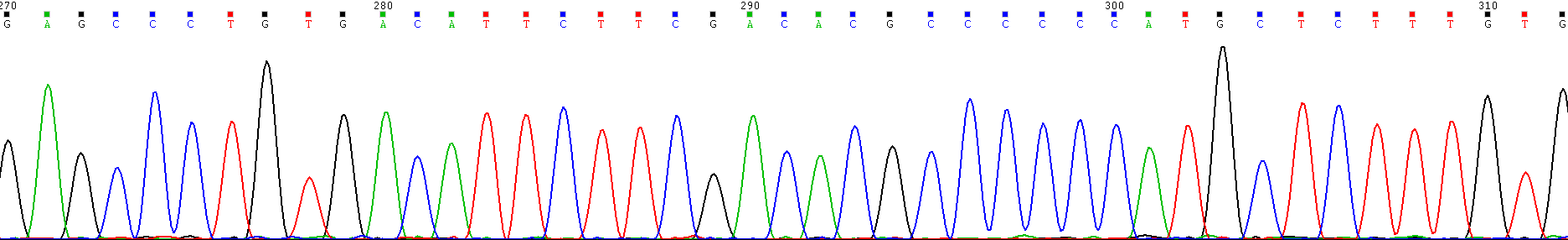 |
| Sequencing of exon 19 (EXON19) |
| 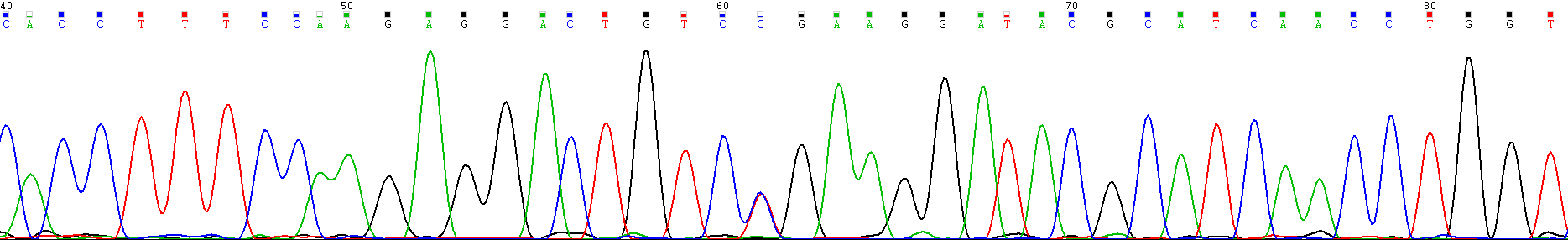 |
| Y. Zhang (father) detected a p.R1319X (c.3955C>T) heterozygous mutation in exon 19 (EXON19). |
| Sequencing of exon 8 (EXON8) |
| 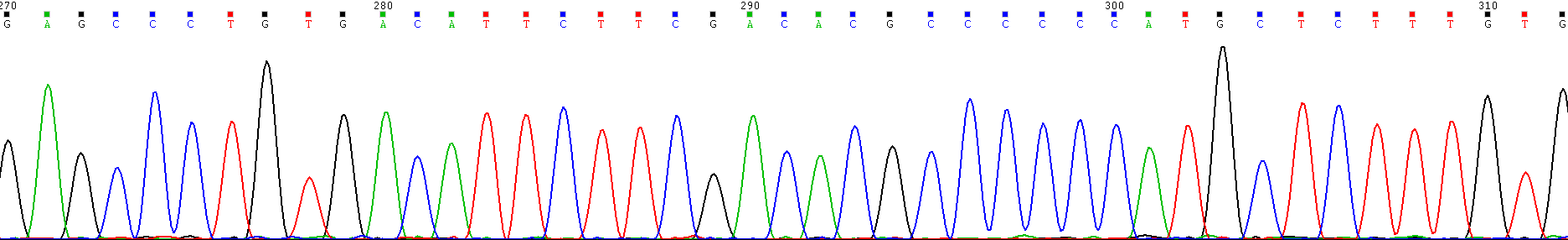 |
| Sequencing of exon 19 (EXON19) |
| 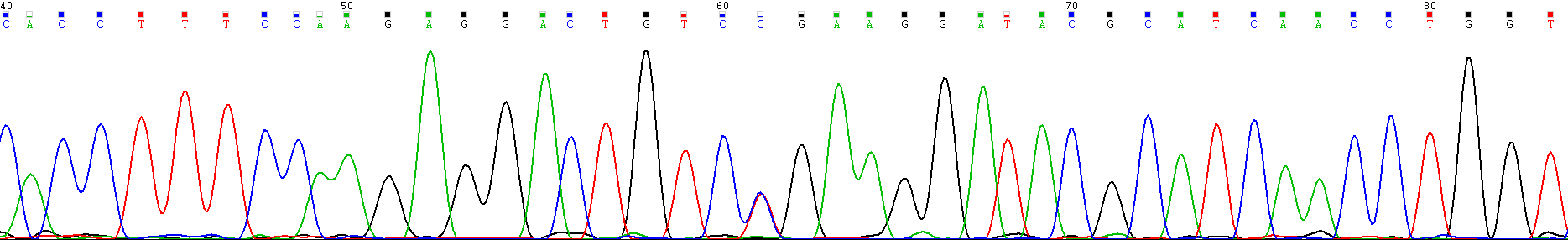 |
| Sun HZ (mother) detected a p.R1319X (c.3955C>T) heterozygous mutation in exon 19 (EXON19). |
